# Evaluation of a Gene-Directed Enzyme-Product Therapy (GDEPT) in Human Pancreatic Tumor Cells and Their Use as In Vivo Models for Pancreatic Cancer

**DOI:** 10.1371/journal.pone.0040611

**Published:** 2012-07-16

**Authors:** Juraj Hlavaty, Helga Petznek, Harry Holzmüller, Angelika Url, Gerrit Jandl, André Berger, Brian Salmons, Walter H. Günzburg, Matthias Renner

**Affiliations:** 1 Institute of Virology, Department of Pathobiology, University of Veterinary Medicine, Vienna, Austria; 2 Christian-Doppler Laboratory for Innovative Immunotherapy, Vienna, Austria; 3 Institute of Pathology and Forensic Veterinary Medicine, Department of Pathobiology, University of Veterinary Medicine, Vienna, Austria; 4 Division of Medical Biotechnology, Paul-Ehrlich-Institut, Langen, Germany; 5 Austrianova Singapore Pte Ltd., Singapore, Singapore; Juntendo University School of Medicine, Japan

## Abstract

**Background:**

Gene-directed enzyme prodrug therapy (GDEPT) is a two-step treatment protocol for solid tumors that involves the transfer of a gene encoding a prodrug-activating enzyme followed by administration of the inactive prodrug that is subsequently activated by the enzyme to its tumor toxic form. However, the establishment of such novel treatment regimes to combat pancreatic cancer requires defined and robust animal model systems.

**Methods:**

Here, we comprehensively compared six human pancreatic cancer cell lines (PaCa-44, PANC-1, MIA PaCa-2, Hs-766T, Capan-2, and BxPc-3) in subcutaneous and orthotopical mouse models as well as in their susceptibility to different GDEPTs.

**Results:**

Tumor uptake was 83% to 100% in the subcutaneous model and 60% to 100% in the orthotopical mouse model, except for Hs-766T cells, which did not grow orthotopically. Pathohistological analyses of the orthotopical models revealed an infiltrative growth of almost all tumors into the pancreas; however, the different cell lines gave rise to tumors with different morphological characteristics. All of the resultant tumors were positive for MUC-1 staining indicating their origin from glandular or ductal epithelium, but revealed scattered pan-cytokeratin staining. Transfer of the cytochrome P450 and cytosine deaminase suicide gene, respectively, into the pancreatic cancer cell lines using retroviral vector technology revealed high level infectibility of these cell lines and allowed the analysis of the sensitivity of these cells to the chemotherapeutic drugs ifosfamide and 5-fluorocytosine, respectively.

**Conclusion:**

These data qualify the cell lines as part of valuable *in vitro* and *in vivo* models for the use in defined preclinical studies for pancreas tumor therapy.

## Introduction

Despite extensive scientific efforts and accumulating knowledge on pancreatic cancer biology, mortality rates of this mostly fatal disease have not been significantly lowered during the last 30 years. Moreover, the worldwide incidence of this disease is increasing [1], [2]. When feasible, the current standard of care involves surgical resection with or without postoperative chemotherapy or chemo−/radiotherapy (for review see [3]). Until recently, the most common chemotherapeutic agent used for treatment of pancreatic cancer is 5-fluorouracil (5-FU), given either alone or in combination with other chemotherapeutic drugs and/or radiotherapy [4], [5], [6]. However, another nucleotide analogue, gemcitabine (Gemzar), brought onto market in 1997 primarily for its palliative effects rather than for improving survival, has rapidly become the chemotherapeutic treatment of choice for pancreatic cancer due to its therapeutic potential alone or in combination [7], [8], [9].

Nevertheless, novel treatment options are urgently required, and during the past decades, a number of investigators have started to evaluate new and unconventional strategies for treatment of pancreatic cancer. This includes novel immunological strategies employing monoclonal antibodies and therapeutic vaccines, as well as gene-based approaches such as antisense nucleic acids, expression of dominant-negative oncogene mutants, expression of tumor suppressor genes or gene-directed enzyme prodrug therapy (GDEPT; for review see [10], [11]). In GDEPT, also known as suicide gene therapy, a specific, heterologous gene is introduced into the tumor cells [12]. When expressed, the respective gene product is able to locally convert a systemically administered non-toxic prodrug into its active cell-toxic form, exerting the therapeutic effect in the tumor cells as well as in surrounding cells due to a so-called bystander effect mediated by diffusion of the toxic metabolites.

Herpes simplex virus thymidine kinase (HSVtk), cytosine deaminase (CD) and cytochrome P450 (CYP) are suicide genes that previously have been shown to be effective in different tumor model systems (reviewed in [13]). Viral vectors based on retroviruses, such as the murine leukemia virus (MLV), have gained significant popularity for efficient and stable gene expression of these suicide genes in tumor cells, thus, several studies employed retroviral vectors for delivery of therapeutic genes into pancreatic tumor cells (for review see [14]).

As a first step to evaluate new ideas and concepts of treatment, experiments involving available human pancreatic tumor cell lines are often performed. Despite the importance of *in vitro* experiments, the interpretation of the results and the conclusions have to be critically evaluated, since the models used often represent an artificial system which may not accurately reflect the *in vivo* situation. The absence of mouse models recapitulating critical elements of the disease has hampered non-clinical studies [15], including those of GDEPT approaches. Therefore, further non-clinical evaluation of new treatment modalities requires the presence of a suitable animal model of human pancreatic cancer. Previously, the development of in vivo pancreatic tumor models has been described by Mohammad and colleagues, who generated a xenograft mouse model (by implantation of primary human tumor material) and animal models using primary patient cells as well as cell lines [16], [17], [18]. Recently, several pancreatic cancer models based on genetically modified animals have been established (for review see [19]).

In the present study, we aimed to analyze and compare six different human pancreatic tumor cell lines in subcutaneous and orthotopic mouse tumor models as well as to evaluate the suitability of these models for non-clinical studies of GDEPT. The subcutaneous tumors were formed in SCID/beige mice using each of the cell lines. In the orthotopic model, five of the six cell lines gave rise to tumors and most of these infiltrated the pancreas with the exception of the Capan-2 cell line. Furthermore, we have been able to show that retrovirus-mediated expression of suicide genes encoding cytosine deaminase or cytochrome P450 2B1 confers the sensitivity of the transduced cells to the respective prodrug (5-fluorocytosine, ifosfamide) at clinically relevant concentrations. The data presented here might be of importance for other researchers with respect to the choice of a suitable animal model of human pancreatic cancer.

## Results and Discussion

In this study, we compared a panel of human pancreatic carcinoma cell lines PANC-1, PaCa-44, Capan-2, BxPC-3, MIA PaCa-2, and Hs-766T in terms of suitability for an *in vivo* mouse model for pancreatic cancer. BxPC-3, Panc-1, PaCa-44 and Capan-2 cell lines were derived from ductal adenocarcinomas of pancreas, whereas MIA PaCa-2 cells originate from carcinomas of the pancreas body [20], [21], [22], [23], [24]. The Hs-766T cell line was derived from the lymph node metastasis of pancreas carcinoma [25]. All cell lines were *in vitro* grown as adherent monolayer cultures with epithelial (PANC-1, MIA PaCa-2, Hs-766T, BxPC-3) or round to polygonal (Capan-2, PaCa-44) morphology. Since the genetic integrities of the tumor markers p53, K-ras, p16 and Smad4 (also known as DPC) are associated with the extent of tumorigenicity [26], we compared the cell lines used in respect to their genotype for these markers (Table 1). No clear consistency in the genetic alterations of the examined genes of the respective cell lines is obvious and the findings are often dependent on the type of analysis applied (for review see [27], [28]). Nearly all cell lines had mutations in the cell cycle-regulating tumor suppressors p53 and p16. Mutations within the pancreatic carcinoma associated tumor suppressor Smad4, which is involved in transforming growth factor beta (TGFβ) signalling, was found impaired only in the cell lines Hs-766T and BxPC-3 [27]. From all used cell lines in this study, only BxPc-3 is described to have an intact K-ras oncogene [27].

**Table 1:** Molecular alterations of tumor-associated markers in human pancreatic tumor cell lines (based on [27], [28]).

	p53	K-ras	p16	Smad4
PANC-1	mut^a^	mut	del^b^	Wt
BxPC-3	mut	wt^c^	wt/del*	Del
PaCa-44	mut	mut	del	Wt
MIA PaCa-2	mut	mut	del	Wt
Hs-766T	mut	mut	wt/mut*	Del
Capan-2	wt	mut	wt/mut*	Wt

a mutated;

b deleted,

c wild type,

* no consistent information available.

### Subcutaneous and Orthotopic Tumor Formation

**Figure 1:**
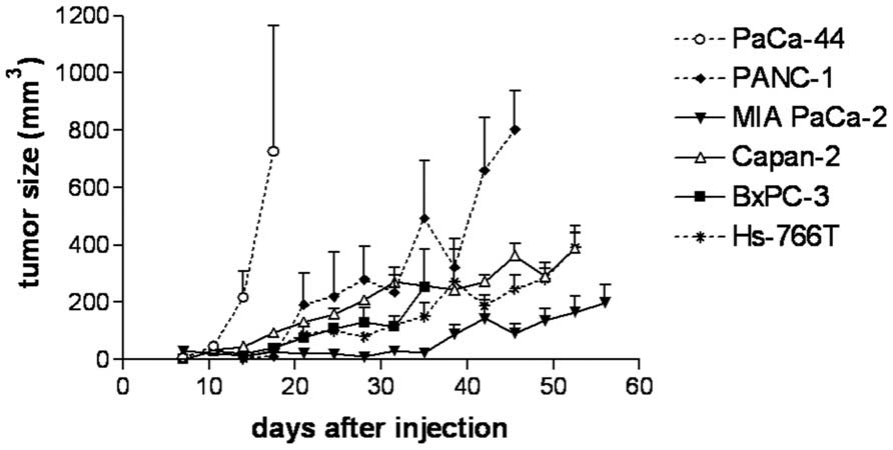
Tumor growth development of subcutaneously growing tumors. 5×10^6^ cells of each pancreatic cell line were subcutaneously injected into the left flank of C.B-17/IcrHsd-*Prkcd^scid^ Lyst^bg^* mice at day 0. Visible tumors were measured twice a week with a caliper and size was calculated by the formula “length×width×width/2”.

Upon subcutaneous injection of the various pancreatic cancer cell lines, all animals showed tumor formation (except one mouse in the PANC-1 group). PaCa-44-derived tumors showed the most rapid but non-homogenous growth. Seven days after cell injection these tumors were measurable with a caliper and the first animal had to be sacrificed after 18 days as tumor size became greater than 1900 mm^3^, thereby having shown a growth rate of more than 1600 mm^3^ within four days (Fig. 1). The second most pronounced tumor growth rate was observed from PANC-1 tumors, followed by Capan-2, BxPC-3 and Hs-766T cells, which displayed a more moderate tumor growth. Similar to PaCa-44, MIA PaCa-2 tumors were measurable within seven days after injection, but in contrast, they maintained their size for 28 days before continuing growing (Fig. 1).

To determine the orthotopical behaviour of the generated pancreas tumors, pieces of the subcutaneously formed tumors were transplanted in close proximity to the pancreas of C.B-17/IcrHsd-*Prkcd^scid^ Lyst^bg^* mice. PaCa-44, PANC-1, BxPC-3 and Capan-2 cells grew intraperitoneally (i.p.) in all animals. MIA PaCa-2 tumor growth was found i.p. in three of the five animals (Table 2). Although Hs-766T tumor pieces were implanted repeatedly i.p., no tumor formation was observed (Table 2), although re-analysis of implanted pieces confirmed tumor cell viability (data not shown).

**Table 2:** Summary of orthotopical tumor growth.

Cell line	Animals (n)	Orthotopic growing tumors	Tumors connected to the pancreas^a^
PaCa-44	6	6	67%
PANC-1	6	6	67%
MIA PaCa-2	5	3	67%
BxPC-3	6	6	83%
Capan-2	6	6	17%
Hs-766T	12	0	**-**

Number of tumors growing after i.p. injection into mice and percentage of tumors that infiltrated the pancreatic tissue.

a infiltrative tumor growth rate into the tail of the pancreas; percentages indicate the proportion of mice with infiltrative tumor growth among those with orthotopic tumor growth.

Over two third of PaCa-44, PANC-1, MIA PaCa-2 and BxPC-3 tumors infiltrated the pancreas. However, the residual tumors had a connection to the abdominal wall instead of to the pancreas (Table 2). From the Capan-2 tumors, only one infiltrated the pancreas, whereas the others were attached to the spleen or to the abdominal wall without showing penetration (Table 2). In contrast to the other cell type, Hs-766T derived tumors were not attached to any organ at all and were found located loosely in the abdomen.

### Tumor Pathology

Tumors of all of the orthotopically growing cell lines as well as the subcutaneously growing cell line Hs-766T were analysed histologically. Analysis of almost all of the orthotopic tumor models revealed focal invasion of the autochtonic pancreas by a solid anaplastic carcinoma except the BxPC-3 tumor model which showed disseminated tubular differentiation and Capan-2 cells which grew as a ductal adenocarcinoma. PANC-1, BxPC-3 and Capan-2 tumors showed little cellular and nuclear pleomorphism and were made up by large cells with abundant pale cytoplasm and large nuclei (PANC-1, Capan-2) or small, round cells with dense nuclei (BxPC-3). PaCa-44 and MIA PaCa-2 tumors, in contrast, had prominent cellular and nuclear pleomorphisms. MIA PaCa-2 tumors mainly consisted of small, round to spindle-shaped cells with dense nuclei besides large cells revealing hyper-eosinophilic cytoplasmic zones typical for acinar cells, while PaCa-44 tumors revealed large, cytoplasm-rich cells with large nuclei. Besides Capan-2 and BxPC-3 tumor cells which had only a moderate rate of mitosis, cells of all other tumors showed a highly increased mitotic activity indicating the high malignancy of these tumors. Furthermore, in PANC-1, MIA PaCa-2 and BxPC3 tumors large areas undergoing necrosis could be observed – a further characteristic feature of highly malignant tumors. Taken together, almost all tumors revealed a high grade of dedifferentiation, with only Capan-2 tumors as well as in some areas of BxPC-3 tumors residual features typical of the glandular origin of the tumors are detectable.

**Figure 2:**
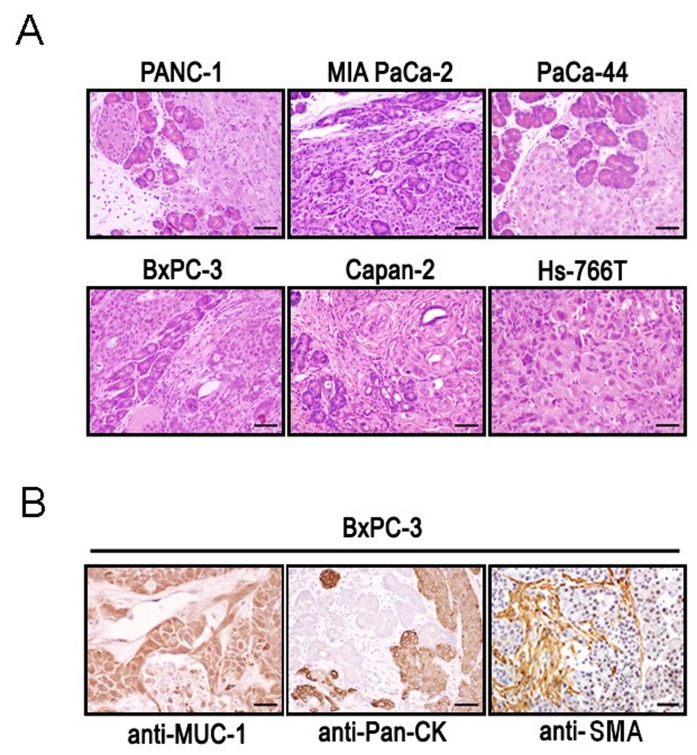
Pathohistological analyses of tumor tissues. (**2A**) Sections of pancreatic tumors derived of the indicated pancreatic cell lines were stained with haematoxylin and eosin. The Hs-766T specimen was dissected from a subcutaneous tumor; all other samples were taken from orthotopically grown tumors. (**2B**) Different sections of a BxPC-3-derived tumor were subjected to immunohistological analysis with antibodies against Mucin-1 (MUC-1), pan-cytokeratin (Pan-CK), and α-smooth muscle actin (SMA). The black bar represents a length of 53 µm, except for anti-MUC-1 staining (27 µm).

Only little lymphocytic and neutrophilic infiltration of the tumor-adjacent tissue, such as adipose and fibrous tissue and the autochtonic pancreas, was detected in the PANC-1, PaCa-44, BxPC-3, MIA PaCa-2, and Capan-2 models with additional lymphocytic infiltration into the PaCa-44 tumor tissue itself (Fig. 2A). Additionally, diffuse moderate neutrophilic invasion of the entire MIA PaCa-2 derived tumors with focal areas of colliquation was observed (Fig. 2A). As orthotopical implantation of Hs-766T tumor pieces did not result in tumor growth, the analogous subcutaneous model was histologically analysed revealing a solid anaplastic carcinoma with prominent cellular and nuclear pleomorphisms, mainly consisting of small, round to spindle-shaped cells with dense nuclei, a high mitotic activity, and large areas of necrotic tumor tissue (Fig. 2A). Broad branches of fibrous tissue were detectable in the periphery of the tumors, but also disseminated adjacent to tumor cell groups in BxPC-3, Capan-2 and PaCa-44 tumors; in PaCa-44 tumors fibrosis was accompanied by immature leukocytic infiltrates.

PAS staining was performed to identify tumor structures originating from ducts and/or acini. In Capan-2 and BxPC-3 tumors 40% and 25% of the tumor cells were positive for PAS, respectively. All other tumors, with the exception of MIA PaCa-2 and PANC-1, which remained completely negative, revealed only a marginal number of PAS-stained cells - most probably due to their more dedifferentiated status (data not shown).

Immunohistochemical (IHC) staining of MUC-1 protein, which is expressed by most glandular and ductal epithelial cells, led to discrete labelling of almost the whole tumor mass in the PaCa-44, PANC-1, BxPC-3 (Fig. 2B) and Capan-2 orthotopic tumor models and of about 75% of the MIA PaCa-2 tumor. Compared with the immunological signals observed within the normal human pancreas, where brownish labelling was found within the cytoplasm of acini and ductal epithelial cells, the expression profiles of those tumors are indicative for their acinar and ductal origin. Although histomorphology only rarely resembles autochtonic pancreas tissue, according to the staining profiles mentioned above, those tumors are identifiable as adenocarcinoma of ductal origin, which accounts for 85% to 90% of all pancreatic tumors [29], [30]. However, according to WHO classification, which recognizes several variants besides the classic tubuloglandular ductal adenocarcinoma [30], almost all of the orthotopic tumors correspond to the anaplastic carcinoma variant. Interestingly, IHC detection of cytokeratins (CK) 1–8, 10, 14–16 und 19 using an Pan-CK antibody resulted in immune labelling of the entire tumor established with PaCa-44, BxPC-3 and Capan-2 cells but in only 60% and 40% stained cells in MIA PaCa-2 and PANC-1 tumors, respectively. As Pan-CK mainly stained tubular/ductal structures of the normal human pancreas, the decreased immunolabelling in MIA PaCa-2 and PANC-1 compared to the other tumors may be suggestive for a more acinar origin of these tumors, or, mainly in case of MIA PaCa-2, which also expressed MUC-1 in only 75% of the tumor cells, the expression pattern may be indicative of a higher degree of malignancy, which is supported by the histological appearance of this tumor with large areas of necrosis, neutrophilic colliquation and a high mitotic rate. The signals obtained by analysing the ability of pan-CK and MUC-1 antibodies to bind to the cell lines were inhomogeneous and inconsistent, which complicates the interpretation of this data.

In summary, according to their histological appearance, the PAS-staining pattern, and the expression profiles for MUC-1 and Pan-CK, MIA PaCa-2 and PANC-1 cell lines gave rise to the most dedifferentiated tumors of this study; however, unequivocal resemblance to healthy human pancreas was given in neither of the tumors, as, with the exception of the small tubular area in Capan-2, even the non-anaplastic tumors showed a solid, non-glandular growth pattern.

Using anti-SMA, numerous deeply brownish coloured cytoplasm rich stromal cells adjacent to tumor cells were found throughout the tumors of all cell lines; normal pancreas remained negative (Fig. 2). Distinct fibrosis accompanied the PSCs, most obviously in BxPC-3, Capan-2 and PaCa-44 tumors. GFAP staining revealed positive immunostaining only in MIA PaCa-2 tumors, representing labelled stellate cell processes surrounding the pancreatic acini. Thus, as activated PSCs are known to be the principle effector cells in fibrosis, which is a consistent pathological feature in human pancreatic cancer [31], [32], [33], and disrupting the desmoplastic tumor stroma enhances delivery and efficacy of anti-tumor drugs [33]. BxPC-3, Capan-2, and PaCa-4 cells represent the most promising tumor models for studies concerning the inhibition of fibrogenesis in human pancreatic cancer.

Our data indicate that, by employing different pancreatic cell lines in an orthotopic setting, a broad spectrum of heterologous pancreatic tumor types could be established serving as model for different therapeutic applications in pancreas tumor therapy. Previous studies in SCID mice revealed that s.c. implanted tumors only rarely show metastasis [16]. In our study, mice were kept for up to 18 weeks after Capan-2 and BxPC-3 tumor onset, 13 and 10 weeks after MIA PaCa-2 and PANC-1 tumor onset, respectively, and 4 weeks after PaCa-44 tumor onset and gross analyses of mouse organs indicate that none of the tumor cells led to local or distal metastases either after s.c. or orthotopic transplantation. In contrast, Tseng and colleagues recently found liver metastases in B6/129 mice by 6 to 8 weeks after implanting cell lines from liver metastases obtained from K-ras(G12D/+), LSL-Trp53(R172H/+) and Pdx-1-Cre mice [34]. As in humans, most if not all pancreas carcinomas show local and distal metastasis over time, the lack of metastatic tendency has to be taken into account when establishing a drug efficacy trial with those tumor cell lines.

In addition to in vivo models carrying human cell xenografts, recently, genetically engineered mouse models of pancreatic cancer have been established (for review see [19], [35]). Since these model systems are based on single genetic modifications, they are valuable tools to study the influence of such genetic alterations or of single signaling pathways on tumor development [19]. However, they are less suitable to evaluate novel therapeutic concepts for pancreatic cancer treatment. Instead, model systems like orthotopically growing human tumors xenografts are far better suited as they resemble, due to the origin of human pancreatic tumors, the current status and the heterogeneity of the human disease.

### Cytosine Deaminase-mediated Sensitivity of Human Pancreatic Cell Lines Towards 5-fluorocytosine

To comparatively study the killing potential of the cytosine deaminase/5-FC system in our pancreatic cancer models, each of the six tumor cell lines was stably infected *in vitro* with the retrovirus vector PCCDWmCMVpuro, harbouring the yeast cytosine deaminase (CD) gene under the transcriptional control of the ubiquitous murine cytomegalovirus (CMV) promoter [36]. The yeast CD allows conversion of the non-toxic prodrug 5-fluorocytosine (5-FC) to the highly toxic chemotherapeutic agent 5-fluorouracil (5-FU) and thus, after integration into the pancreatic cancer cells, its expression should allow preferential tumor cell depletion upon 5-FC administration.

**Figure 3:**
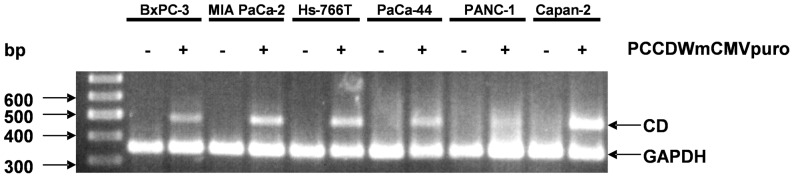
Cytosine deaminase gene expression in PCCDWmCMVpuro-transduced and in parental cell lines. Total cellular RNA was isolated from indicated cell lines and mRNA was reverse transcribed using oligo(dT) primers. CD- as well as GAPH-specific gene fragments were amplified using specific primers and separated on an agarose gel showing the expected sizes of ∼480 bp for CD and ∼350 bp for GAPDH. The cDNA amount used for PCR amplification was adjusted to yield comparable amounts of the GAPDH gene fragment.

To confirm CD transcription after retrovirus transduction, RT-PCR was performed from lysates of transduced cells as well as from non-transduced cells. The ∼480 bp CD gene fragment was clearly detected in cells transduced with vector PCCDWmCMVpuro (Fig. 3), but was absent in non-infected cells. These data indicate proper vector integration and comparable expression of the CD transgene in all cell lines (Fig. 3).

To assess this GDEPT strategy for treatment of pancreatic cancer cells *in vitro*, we determined the LD_50_ after administration of the prodrug 5-FC to human pancreatic tumor cell lines previously transduced with PCCDWmCMVpuro. Compared to untransduced cells that were not responsive to 5-FC (>1000 µg/ml), the LD_50_ was reduced by 16-fold on average in all cell lines (Table 3). This eminent increase of 5-FC sensitivity indicates that expression of CD successfully established conversion of the prodrug 5-FC to the toxic 5-FU in all transduced pancreas tumor cell lines. Interestingly, the CD expressing cell line BxPc-3 was up to 30-fold more sensitive to 5-FC compared to transduced Hs-766T cells. To analyze whether this difference in sensitivity is independent of CD expression, both untreated and transduced pancreatic cell lines were directly incubated with toxic 5-FU. As expected, the susceptibility of all cell lines to 5-FU was independent of transduction with vector PCCDWmCMVpuro. However, in accordance to 5-FC treatment results, BxPC-3 cells were about 30-fold more sensitive to 5-FU than Hs-766T cells (Table 3), suggesting that the tested pancreatic cell lines have a differential susceptibility to the toxic drug *per se*. According to clinical studies employing 5-FC, plasma concentrations in patients are reaching levels of 50–125 µg/ml 5-FC after oral administration of 150 mg 5-FC per kg body weight [37], [38], [39]. However, plasma concentrations above 100 µg/ml are often considered as toxic for the patient [39]. In this respect, treatment with vector PCCDWmCMVpuro allowed four out of six cell lines tested to respond to 5-FC concentrations below 100 µg/ml, suggesting that GDEPT may allow an efficacious tumor treatment at prodrug doses below the levels of non-specific toxicity.

**Table 3:** Sensitivity of human pancreatic cancer cell lines towards 5-FC and 5-FU.

Cell line	5-fluorocytosine (LD_50_, µg/ml)		5-fluorouracil (LD_50_, µg/ml)	
	mock	CD	mock	CD
PANC-1BxPC-3PaCa-44MIA PaCa-2Hs-766TCapan-2	>1000>1000>1000>1000>1000>1000	3.34±0.642.97±9.52119.76±27.4518.15±8.89120.32±68.1458.43±11.01	0.26±0.115.29±2.595.53±2.891.39±0.970.78±0.130.99±0.7	0.13±0.031.17±1.066.33±3.660.97±0.321.1±0.714.05±2.53

Cells stably transduced with the yeast CD gene as well as non-transduced parental cells (mock) were cultured in increasing concentrations of 5-FC or 5-FU for five days as described in Materials and Methods. LD_50_ values were calculated using Excel Fit software. Each experiment was performed in quadruplicates. Shown are data from three independent experiments.

### Cytochrome P450-mediated Killing of Human Pancreatic Tumor Cell Lines

The use of the chemotherapeutic agent ifosfamide for the treatment of pancreatic cancer has been described in several studies [40], [41], [42], [43], [44]. The prodrug ifosfamide is converted to phosphoramide mustard by cytochrome P450. Phosphoramide mustard renders DNA of dividing cells dysfunctional via alkylation, which is the basis for its selectivity for dividing cells such as comprised in fast-growing tumors. Unfortunately, as cytochrome P450 is predominantly produced in the liver, toxicity of the ifosfamide metabolite is rather systemic and not tumor-specific. To enhance tumor-specific toxicity in our comparative pancreas tumor model, we used the retroviral vector PCCWmCMV harbouring the rat cytochrome P450 2B1 (CYP2B1) gene for stable transduction of the pancreatic cell lines.

**Figure 4:**
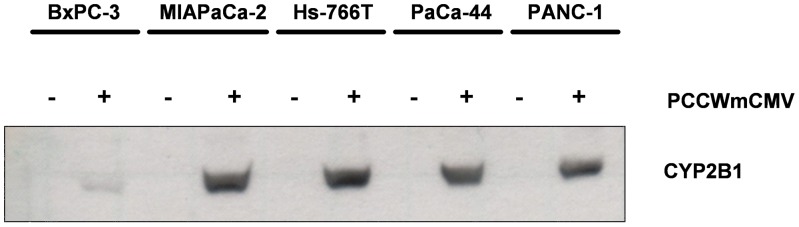
Cytochrome 2B1 protein expression in PCCWmCMV-transduced and non-transduced parental cell lines. Total cellular lysates from 8×10^5^ cells were separated on a 10% polyacrylamide gel under denaturing conditions. After blotting, CYP2B1 protein was detected with a CYP-specific antibody.

To monitor expression of CYP2B1 after transduction, Western blot analyses have been performed with protein lysates of transduced and non-transduced cells. CYP2B1 expression was only detected in cells previously transduced with the retroviral vector (Fig. 4). Cell line BxPC-3 revealed only minor expression levels of CYP2B1. To evaluate the sensitivity of the CYP2B1-expressing pancreatic tumor cell lines BxPC-3, MIA PaCa-2, Hs-766T, PaCa-44 and PANC-1 towards ifosfamide, the LD_50_ was determined in comparison to untransduced cells. The unspecific toxicity of the prodrug ifosfamide in non-transduced cell lines varied significantly and was ranging from 0.18 mM (Hs-766T cells) up to 2 mM (PANC-1 cells) LD_50_ values (Table 4). Expression of the CYP2B1 transgene led to an up to 13-fold increased susceptibility to ifosfamide in four out of the five cell lines tested (Table. 4). Only Hs-766T cells, which were most sensitive to ifosfamide *per se*, showed no enhanced susceptibility to ifosfamide in spite of CYP2B1 gene expression.

**Table 4:** Sensitivity of human pancreatic cancer cell lines towards ifosfamide.

Cell line	Ifosfamide (LD50, mM)	
	Mock	CYP2B1
PANC-1BxPC-3PaCa-44MIA PaCa-2Hs-766T	2.00±0.121.40±0.101.45±0.120.75±0.090.18±0.08	0.16±0.010.56±0.040.22±0.020.17±0.030.18±0.06

Cells stably transduced with the CYP2B1 gene as well as non-transduced parental cells (mock) were cultured in increasing concentrations of ifosfamide for five days as described in Materials and Methods. LD_50_ values were calculated using Excel Fit software. Each experiment was performed in quadruplicates. Data from three independent experiments are shown.

Studies on the feasibility of in vivo GDEPT for pancreatic cancer have already been performed previously [44], [45], [46], [47], [48]. We have shown that encapsulated cells genetically modified to express the cytochrome P450 suicide gene implanted in mice in the vicinity of tumors derived from PaCa 44 cells are able to cause tumor reduction, when the appropriate prodrug that is activated by cytochrome P450 is given [45], [46]. In addition, we have used these encapsulated cells in a clinical trial in patients with pancreatic cancer and were able to show that the median survival of this patient group was doubled [44], [47].

Taken together, these data suggest that such GDEPT approaches for the treatment of pancreatic cancer are promising. However, such novel therapies need to be tested in more than one relevant animal model system resembling the characteristic of the respective tumors as far as possible. This clearly indicates an urgent need for further characterisation of animal models of choice, as we have shown in this study.

## Materials and Methods

### Ethics Statement

All animals were handled in strict accordance with good animal practice. Animal work was approved by the Advisory Committee for Animal Experiments of the Federal Ministry of Education, Science and Culture according to the Austrian Federal Law of Animal Experiments (BGBl No. 501/1988, BGBl I No. 169/1999, and BGBl I No. 136/2001) and a license for animal testing was issued specifically for this study (GZ 68.205/177-BrGT/2003). All efforts were made to minimize animal suffering. Surgeries were performed under general anesthesia. Animals were killed before the tumor reached a palpable size of 1500 mm^3^.

### Plasmids

The coding sequence of the cytosine deaminase gene (CD, GenBank accession no. U55193, pos. 183–659) from *Saccharomyces cerevisiae* was PCR amplified from yeast genomic DNA (Sigma) and subsequently introduced into the pCR-XL-TOPO cloning vector (Invitrogen) resulting in plasmid pYCD. Then, the cytosine deaminase cDNA was released from plasmid pYCD via an *EcoR*I restriction digest and ligated with the 7.7 kb *EcoR*I-fragment of plasmid pPCEWmCMVpuro encoding an MLV-based retroviral vector [36]. The resulting plasmid was named pPCCDWmCMVpuro. The coding sequence of the rat cytochrome P450 2B1 (CYP2B1) gene was PCR amplified from plasmid pc3/2B1 [47] using primers Age-CYP (5′-GCGTACCGGTCCACCATGGAGCCCAGTATCTTGC-3′) and CYP-Not (5′-AAGCGGCCGCTATCACCGAGCTGAGAAGCAG-3′) harboring *Age*I and *Not*I specific restriction sites, respectively, and cloned into the large *Age*I-*Not*I fragment of the retroviral vector plasmid pPCEmCMV [36]. The resulting plasmid was named pPCCmCMV. To generate plasmid pPCCWmCMV, the woodchuck hepatitis virus posttranscriptional regulatory element (WPRE) was released from plasmid pPCEWmCMV by restriction digest with *Not*I and inserted into the *Not*I-linearised vector pPCCmCMV [36].

### Cell Lines

Amphotropic retroviral packaging cells based on the human embryonic kidney cell line HEK293 [49], human pancreatic tumor cell lines PANC-1 (ATCC CRL-1469), PaCa-44 [22], MIA PaCa-2 (ATCC CRL-1420), and Hs-766T (ATCC HTB-134) were grown in Dulbeccós modified Eaglés medium (DMEM/Glutamax; Life Technologies) supplemented with 10% foetal calf serum (FCS, Life Technologies). Human pancreatic tumor cell lines BxPC-3 (ATCC CRL-1687) and Capan-2 (ATCC HTB-80) were cultivated in RPMI medium (Life Technologies) supplemented with 10% FCS and glutamine and McCoýs medium (Life Technologies) supplemented with 10% FCS, respectively. NIH3T3 cells (ATCC CRL-1658) were maintained in DMEM/Glutamax supplemented with 5% FCS.

### Virus Production and Generation of Stably Infected Cells

The calcium phosphate co-precipitation method was used to establish stably virus producing cells [50]. Briefly, 7×10^5^ of HEK293 based retroviral packaging cells [49] were transfected in a 6-well plate with 3.0 µg of plasmid pPCCDWmCMVpuro and pPCCWmCMV, respectively. 24 hours later, cells were trypsinised and selected in DMEM containing either 0.6 µg/ml Puromycin (Sigma) for pPCCDWmCMVpuro transfected cells or 400 µg/ml Geneticin (Gibco) for pPCCWmCMV transfected cells until single cell colonies were formed. Selected cells were expanded and used for further experiments. To generate stably infected pancreatic tumor cells, 2×10^5^ PANC-1, PaCa-44, MIA PaCa-2, Hs-766T, Capan-2 and BxPC-3 cells per well of a 6-well plate were plated the day before infection. Infection was carried out in the presence of polybrene (Sigma, 8 µg/ml) using 1 ml of a 10-fold diluted virus-containing supernatant obtained from stably PCCDWmCMVpuro and PCCWmCMV virus producing cells, respectively. The infected cells were trypsinised 24 hrs after infection and selected in DMEM containing selection antibiotics as described above.

### RT-PCR and Western Blot Analysis

Total cellular RNA from cells in logarithmic growth was isolated using the RNeasy Mini Kit (Qiagen) according to the manufacturer’s recommendations. cDNA was synthesised using the SuperScript™ II RNaseH^−^ Reverse Transcriptase Kit (Invitrogen). Amplification of the CD cDNA and a cDNA fragment of the housekeeping gene glycerol-aldehyde-3-phosphate dehydrogenase (GAPDH) was done using standard PCR techniques. Briefly, 1 µg of the total cellular RNA was reverse transcribed using oligo dT primer. The CD gene fragment as well as the GAPDH gene fragment were amplified using specific primers (CDf 5′-GCAATCATGGTGACAGGGGGAATG-3′ and CDr 5′-CTACTCACCAATATCTTCAAACC-3′ for the CD gene and hGAPDEg 5′-ATGCCTCCTGCACCACCAAC-3′ and hGAPDHgr 5′-CGCCTGCTTCACCACCTTCT-3′ for the GAPDH gene). PCR consists of an initial denaturation step at 94°C for 60 sec followed by 30 cycles of denaturation at 94°C for 30 sec, annealing at 58°C for 30 sec and elongation at 72°C for 40 sec (in the case of GAPDH) or 60 sec (in the case of CD). The final elongation was performed at 72°C for 10 minutes. The cDNA amount used for PCR amplification was adjusted in a way yielding comparable amounts of the housekeeping GAPDH gene fragment. The same amounts of the cDNA were also used for CD amplification.

For Western blot analysis, total cellular lysates were prepared by lysis of 4×10^6^ cells in 100 µl sample buffer (130 mM Tris-HCl pH 6.8, 10% SDS, 20% glycerol, 10% β-mercaptoethanol, 0.2% bromophenol blue) followed by sonication of the lysed cells. Proteins were separated under denaturing conditions on a 10% polyacrylamide gel and transferred onto a Hybond-P PVDF membrane (Amersham Bioscience). Blots were first incubated with a goat anti-CYP2B1 antibody (Daichii, dilution 1∶5,000), washed, and incubated with a secondary mouse anti-goat IgG antibody coupled to HRP (Daichii, dilution 1∶15,000). Signals were detected using the enhanced chemiluminescence system (ECL, Amersham Biosciences).

### In vitro Cytotoxicity and Resorufin Assays

Cells were plated at a density of 1×10^3^ (PANC-1, PaCa44, Hs-766T) or 5×10^3^ (MIA PaCa-2, Capan-2, BxPC-3) cells per well of a 96-well flat-bottomed plate. The next day, cells were cultured in different concentrations of 5-fluorocytosine (Sigma, 5-FC) or 5-fluorouracil (Sigma, 5-FU) in 100 µl of fresh cell culture medium. Cell viability was measured after five days of incubation using the Cell Proliferation kit II (XTT; Roche) according to manufactureŕs recommendations. The LD_50_ values were calculated using the XLfit software (ID Business Solutions). To analyse the effect of ifosfamide on CYP2B1 expressing pancreatic tumor cells, 5×10^4^ cells were seeded into a 3-cm dish. After overnight incubation in the corresponding medium, ifosfamide was added to a final concentration of 0.25 mM, 0.5 mM, 0.75 mM, 1 mM, and 2 mM. After 5 additional days, the cells were trypsinised and an aliquot mixed with the same amount of 0.4% trypan blue in phosphate buffered saline (PBS, Life Technologies), incubated for 1 min at room temperature, the living, unstained cells were counted using a microscope, and the LD_50_ values calculated.

To determine the CYP2B1 enzymatic activity, 1×10^5^ cells were seeded in a well of a 96-well plate. One day later, 7-pentoxyresorufin as CYP2B1 substrate was added to a final concentration of 15 µM and the cells were incubated for 4 h at 37°C. Using an excitation wavelength of 520 nm, resorufin formation was determined measuring the fluorescence of the samples at 590 nm in a microplate reader (Tecan). Fluorescence of resorufin standards (Sigma) were used for calculation.

### In vivo Experiments

For the evaluation of subcutaneous tumor growth, pancreatic tumor cells were harvested at logarithmic growth and washed twice with PBS. Five×10^6^ cells were injected subcutaneously into the left flank of 8–10 week old C.B-17/IcrHsd-*Prkcd^scid^ Lyst^bg^* mice (Harlan) in a total volume of 100 µl. Mice were examined for tumor growth twice a week. As soon as tumors were palpable, they were measured with a calliper and tumor size was calculated using the formula “length×width×width/2”. For orthotopic implantation of tumors, subcutaneously grown tumors were resected aseptically and cut into 1 mm^3^ pieces which were kept in DMEM/10% FCS supplemented with 100 U/ml penicillin and 100 U/ml streptomycin until implantation. Mice were anaesthetised using 100 mg/kg body weight (BW) ketamine (Graeub) and 4 mg/kg BW xylazine (Intervet). A small incision was made on the left side parallel to the costal arch and the left part of the pancreas was carefully exposed. A tumor piece was placed onto the uneven surface of the pancreas without fixing it. The abdominal wall and the skin were closed in two layers using synthetic absorbable 4-0 surgical suture (SMI). Animals were fed ad libitum with autoclaved laboratory rodent diet (Altromin-R+M-H-1324).

### Tumor Histology and Immunohistochemistry

Orthotopically growing tumors were excised at a maximum size of approximately one centimeter in diameter, fixed in 7% neutral-buffered formalin, embedded in paraffin and cut into 3 µm sections. Tumor sections were routinely stained with hematoxylin and eosin (HE) and for identification of pancreatic acini and ductal structures periodic acid Schiff (PAS) staining was performed. Immunohistochemistry (IHC), using the avidin-biotin-complex technique was applied to formalin-fixed and paraffin embedded tumor sections. Briefly, deparaffinized and rehydrated sections were incubated with 1.5% H_2_O_2_ in methanol for blocking of endogenous peroxidase activity. To reduce background staining the sections were incubated with 10% normal goat serum for 1 h at room temperature in a humidified chamber and for antigen demasking, sections were pre-treated with protease (Sigma). Incubation with the primary antibodies (polyclonal anti MUC-1 antibody, Labvision Neomarkers, dilution 1∶50; monoclonal anti pan-cytokeratin AE1/AE3 antibody, Boehringer Mannheim, dilution 1∶500) was performed overnight at 4°C in a humidified chamber. After extensive washing with phosphate-buffered saline, the sections were incubated with appropriate biotinylated secondary antibodies for 30 min at room temperature in a humidified chamber. Consecutive steps for avidin-biotin-complex binding and visualization of positive reaction products were performed according to the manufactureŕs instructions (Vectastain® ABC-kit and Peroxidase Substrate kit DAB, Vector Laboratories). Samples from human breast carcinoma for MUC-1 staining and from human gut for pan-cytokeratin staining as well as normal human pancreas for both antibodies served as positive controls. To detect stellate cells, anti-glial fibrillary acidic protein (GFAP, DAKO, Denmark, dilution 1∶7000) specific for their quiescence state and anti-α-smooth muscle actin (SMA, DAKO, Denmark, dilution 1∶1500) for activated pancreas stellate cells (PSCs) were applied automatically (Autostainer 360, Lab Vision/Thermo Fisher Scientific, USA).
